# Association between depression or anxiety symptoms and immune-inflammatory characteristics in in-patients with tuberculosis: A cross-sectional study

**DOI:** 10.3389/fpsyt.2022.985823

**Published:** 2022-10-20

**Authors:** Xiangmin Liu, Xinyu Bai, Rong Ren, Lu Tan, Ye Zhang, Huizhen Lan, Qianlan Yang, Jianqing He, Xiangdong Tang

**Affiliations:** ^1^West China School of Nursing, Department of Respiratory and Critical Care Medicine, West China Hospital, Sichuan University, Chengdu, China; ^2^Sleep Medicine Center, Mental Health Center, Translational Neuroscience Center, West China Hospital, Sichuan University, Chengdu, China; ^3^Ward 3, Department of Tuberculosis, The Fourth People Hospital of Nanning, Guangxi Zhuang Autonomous Region, Nanning, China

**Keywords:** tuberculosis, depression, anxiety, immune, inflammation

## Abstract

**Background:**

Depression and anxiety are major psychological issues among patients with tuberculosis (TB) owing to chronic and complex treatments, have been reported to be closely correlated with immune and inflammation. However, the association of peripheral immune-inflammatory characteristics with depression/anxiety symptoms in in-patients with TB has rarely been reported.

**Methods:**

A cross-sectional study of 338 in-patients with TB from 3 hospitals in China were enrolled to investigate their depression and anxiety status by using the nine-item Patient Health Questionnaire (PHQ-9) and seven-item Generalized Anxiety Disorder Scale (GAD-7). Participants were divided into groups based on their PHQ-9 and GAD-7 scores, and differences in demography and immune-inflammatory characteristics were studied. Logistic analysis was performed to explore factors related to depression and anxiety symptoms.

**Results:**

Depression and anxiety prevalence among patients with TB was 47.9 and 42.6%, respectively. Furthermore, 38.5% of patients reported a comorbidity of depression and anxiety symptoms. The counts of CD3, CD4, CD8, and lymphocytes decreased, whereas those of neutrophils, platelets, and peripheral blood cells and their derived indices increased among TB patients with depression or anxiety in comparison with those without symptoms (*p* < 0.05). In addition, increasing age, lower income (monthly income ≤ 3,000 yuan), divorced or widowed, drug resistance, and higher systemic immune inflammation index (SII) were significantly associated with depression or anxiety symptoms (*p* < 0.05).

**Conclusion:**

Approximately half of the patients with TB suffered from depression or/and anxiety symptoms. Patients with depression or anxiety present worse cell immune status and stronger inflammatory responses compared to those without symptoms. We emphasized the importance of paying attention to the dysfunction of immune-inflammation process of TB patients with depression or anxiety symptoms. Especially, SII has a potential application value in guiding the evaluation of TB-related depression or anxiety owing to its easily accessibility and being economical.

## Introduction

Tuberculosis (TB) is a chronic inflammatory disease with highly contagious nature (1). It is the primary cause of death due to single pathogen infection, with a mortality rate of 15% in 2020 (2). Several challenges such as long treatment periods, great side effects, and drug resistance lead to a high prevalence of mental disorders in TB treatment. Previous studies have demonstrated that more than half of the patients with TB possibly suffer from depression and anxiety (3). TB with comorbidity of mental disorders is more prone to poor medication adherence and delayed treatment (4), resulting in higher drug resistance, risk of treatment failure, mortality, and transmission (5, 6). Therefore, the mental health of TB patients should be emphasized.

Having TB affects the human immune system as it can disarm the host immune responses, manifested by abnormalities in the number and function of antigen-specific T-cells (7, 8). Protection against *Mycobacterium tuberculosis* (*Mtb*) is primarily dependent on cell-mediated immunity, particularly that conferred by CD4 and CD8 T cell subsets (8). An obvious decrease of percentage or absolute counts of CD3, CD4, and CD8 T cells in the peripheral blood of patients with TB indicates significant immunosuppression or immune deterioration which is closely related to the severity of TB illness (9). CD4 and CD8 were detected to be lower among college students with anxiety and depression than in those without symptoms, indicating lower concentrations of immune parameters are associated with depression and anxiety symptoms among healthy adults (10).

Accumulating evidence suggests that immune system play an important role in pathophysiology of psychiatric disorders, involving altering immune cell and inflammatory cytokines, neurotransmitter metabolism, neuroendocrine function, neural plasticity, or reactive oxygen species (11). Inflammation is considered a central component of the innate immune response (12). Numerous studies have shown that many psychiatric disorders, such as depression and anxiety, are closely related to inflammation (13, 14). Pro-inflammatory cytokines in infectious diseases can induce behavioral symptoms referred to as sickness behavior, which is accompanied by depression, anxiety or sleep disorder (15). Because of their value and convenience, peripheral blood cell count-derived indices such as neutrophil/lymphocyte ratio (NLR), monocyte/lymphocyte ratio (MLR), platelets/lymphocyte ratio (PLR), and the systemic immune-inflammation index (SII) have been recognized as biomarkers to assess inflammatory sensitivity in recent years (16). Significant research has established these ratios as severity markers in many psychiatric disorders (17), and these indices have been linked to morbidity, mortality, and prognosis in psychiatric and non-psychiatric diseases such as diabetes mellitus and cancers (18–20).

Although several surveys have reported immune-inflammatory abnormality in patients with TB (21, 22), its relationship with mental and emotional health has not been determined completely. Thus, this study aimed to investigate the immune-inflammatory characteristics of TB patients with depression and anxiety.

## Materials and methods

### Study design and settings

A cross-sectional clinical study was conducted at West China Hospital, Guangyuan Mental Health Center, and the Fourth People's Hospital of Guangxi in China from November 2021 to February 2022 using a convenience sampling method. A self-designed demographic questionnaire was used to collect demographic information. Appropriate clinical scoring instruments were used to evaluate their depression and anxiety symptoms. Fasting venous blood samples were collected on the next morning of admission to further have immune and inflammatory test. The Biomedical Research Ethics Committee of West China Hospital, Sichuan University approved this study.

### Participants

Inclusion criteria for the study were as follows: (1) confirmed and highly suspected TB patients who have been diagnosed in accordance with national TB program guidelines (23, 24); (2) those who could complete the questionnaire with their communication and cognitive skills; and (3) those who voluntarily participated in the study. Patients with severe somatic disorders, including but not limited to tumors, critical infections, immune diseases; or confirmed psychiatry disorders; or those took on special drugs, such as psychotropic or immune-altering medications, were excluded.

### Measurements

#### Clinical evaluation

An electronic structured questionnaire was used to collect information containing age, gender, height, weight, education years, marital status, income per month, duration of illness, and drug resistance status. Drug resistance was defined as drug susceptibility testing showed that *Mtb* were resistant to at least one first-line anti-TB drug such as isoniazid, rifampicin.

#### Evaluation of depression and anxiety symptoms

The nine-item Patient Health Questionnaire (PHQ-9) and seven-item Generalized Anxiety Disorder Scale (GAD-7), which are validated and widely applied self-report scales were used to assess depression and anxiety, with an excellent Cronbach's alpha of 0.901 and 0.938, respectively, in the present study. The optimal and most common cut-point of PHQ-9 and GAD-7 used for screening for depressive and anxiety disorders was 10 or above (25, 26). Hence, PHQ-9 and GAD-7 scores above 10 were considered a sign of depression or anxiety in the present study (27).

#### Immune-inflammatory parameters

Data of immune-inflammatory parameters were derived from the hospital information system containing diagnosis, prescriptions, laboratory tests, including T cell subsets, and blood routine examinations. The immune biomarkers, including the CD3, CD4, CD8 counts and the CD4/CD8 ratio. The parameters from blood routine test were used to calculate the inflammatory indicators, including neutrophil/lymphocyte ratio (NLR), monocyte/lymphocyte ratio (MLR), platelets/lymphocyte ratio (PLR), and the systemic immune-inflammation index (SII, SII = platelets X neutrophils/lymphocytes) (28).

### Statistical analysis

Continuous variables were expressed as means ± standard deviation (SD) for normally distributed data or median (interquartile range, IQR) for non-normal distributed data, and group comparisons were performed using the Independent sample's *t*-test or Mann-Whitney U-test, as appropriate. Categorical variables were expressed as frequencies and percentages, and group differences were examined using the Chi square test. A logistic regression analysis was conducted to identify risk factors for depression and anxiety. A statistically significant *p*-value of < 0.05 was used. For statistical analyses, SPSS (version 21.0) was used.

## Results

### Prevalence and demographic characteristics

A total of 350 TB patients were recruited to complete a questionnaire. Of these, 12 questionnaires were deleted due to logical errors or a large number of missing data, with a participation rate of 96.6%. Finally, 338 participants were enrolled with a mean age of 53.8 ± 18.2 years (range: 14–89), comprising 213 men (63%) and 125 women (37%). The average PHQ-9 and GAD-7 scores were 11.7 ± 6.5 and 10.0 ± 5.8, respectively. Using a cut-off score of 10 points, 162 (47.9%) patients exhibited depression scores >10, while 144 (42.6%) patients exhibited anxiety scores >10. In addition, 130 patients (38.5%) exhibited depression and anxiety scores >10. Considering the sample size in the depression only (*N* = 30) or anxiety only (*N* = 14) groups was insufficient for informative analyses, patients were divided into two groups based on PHQ-9 and GAD-7 scores, including those with depression or anxiety (DA) and those without depression or anxiety (DA) (neither depression nor anxiety, NDA).

We first analyzed age, education, body mass index (BMI), sex, marital status, monthly income, duration of illness, and whether there was drug resistance. Except for sex and BMI, there were significant statistical differences between the two groups in all variables. The age of participants in group DA was significantly older than those in group NDA (*p* < 0.05). The education years of participants in group DA were significantly fewer than those in group NDA (*p* < 0.05). The present study also indicated a higher depression or anxiety prevalence among patients with TB who were divorced or widowed, with lower income (monthly income ≤ 3,000 yuan), who were suffering from illness for >12 months, or who were drug-resistant. The details were presented in Table 1.

**Table 1:** Demographic characteristics in patients with tuberculosis.

**Item**	**Without depression or anxiety** **(*N* = 162)**	**With depression or anxiety** **(*N* = 176)**	**F/*χ^2^***	***P***
Age, years	48.6 ± 18.6	58.5 ± 16.4	−5.165	< 0.001
Male, n (%)	95 (58.6%)	118 (67.0%)	2.556	0.110
BMI, kg/m^2^	20.6 ± 3.3	20.2 ± 3.2	1.065	0.287
Education, years	9.7 ± 4.0	8.6 ± 3.7	2.775	0.006
Marital status, *n* (%)				
Unmarried	28 (17.3%)	14 (8.0%)	11.376	0.003
Married	126 (77.8%)	140 (79.5%)		
Divorced or widowed	8 (4.9%)	22 (12.5%)		
Monthly income, *n* (%)				
≤ 3,000	61 (37.7%)	104 (59.1%)	18.311	0.000
3,000–5,000	79 (48.8%)	48 (27.3%)		
>5,000	22 (13.6%)	24 (13.6%)		
Duration of illness, *n* (%)				
≤ 12 months	112 (69.1%)	102(58.0%)	4.540	0.033
>12 months	50 (30.9%)	74 (42.0%)		
Drug resistance, *n* (%)	3 (1.9%)	19 (10.8%)	11.088	0.001

BMI, body mass index; *P* < 0.05.

### The immune-inflammatory characteristics

We secondly analyzed T cell subsets, inflammatory cell counts and their derived indices of the participants. Significant statistical differences were detected in the counts of CD3, CD4, CD8, neutrophil, platelets, lymphocyte, and derived indices between the two groups. The counts of CD3, CD4, CD8, and lymphocytes in the DA group were significantly lower than that in the NDA group (*p* < 0.05). The DA group had significantly higher counts of neutrophils, platelets, and derived indices (NLR, MLR, PLR, and SII), as well as a higher CD4/CD8 ratio than the NDA group (*p* < 0.05). However, no significant differences in monocyte counts were found. The parameters were shown in Table 2.

**Table 2:** Immune-inflammatory parameters in patients with tuberculosis.

**Item**	**Without depression or anxiety** **(*N* = 162)**	**With depression or anxiety** **(*N* = 176)**	**t/Z**	***P***
T lymphocyte counts				
CD3, cell/μl	917.6 ± 440.0	677.7 ± 403.2	5.231	< 0.001
CD4, cell/μl	516.1 ± 257.8	400.2 ± 240.6	4.276	< 0.001
CD8, cell/μl	365.5 ± 258.4	250.5 ± 181.6	3.762	< 0.001
CD4/CD8	1.7 ± 0.8	1.9 ± 1.1	−2.119	0.035
Inflammatory cell				
Neutrophil, 10^9^ cells/L	4.3 ± 2.4	6.4 ± 3.3	−3.646	< 0.001
Monocyte, 10^9^ cells/L	0.5 ± 0.6	0.6 ± 0.4	−1.493	0.136
Platelets, 10^9^ cells/L	270.2 ± 114.3	318.2 ± 140.0	−3.437	0.001
Lymphocyte, 10^9^ cells/L	1.5 ± 0.9	1.0 ± 0.5	5.604	< 0.001
Inflammatory indices (IQR)				
NLR	2.6 (2.0)	6.3 (6.0)	−9.411	< 0.001
MLR	0.4 (0.2)	0.6 (0.5)	−7.614	< 0.001
PLR	192.1 (140.8)	317.5 (333.6)	−7.328	< 0.001
SII	667.9 (652.2)	1878.9 (2281.8)	−9.405	< 0.001

NLR, neutrophil/lymphocyte ratio; MLR, monocyte/ lymphocyte ratio; PLR, platelets/lymphocyte ratio; SII, platelets X neutrophils/lymphocytes; IQR, interquartile range; *P* < 0.05.

### Logistic regression analysis of associated factors in TB patients with depression or anxiety

Logistics regression analysis indicated that increasing age, absence of spouse (divorced or widowed), lower income (monthly income ≤ 3,000 yuan), drug resistance, and higher SII were significantly correlated to the depression and/or anxiety symptoms (*p* < 0.05) (see Table 3).

**Table 3:** Logistic regression analysis of associated factors in tuberculosis patients with depression or anxiety.

**Item**	**With depression or anxiety**		
	**OR**	**95% CI**	***P***
Age	1.038	1.018–1.060	0.000
Male	1.004	0.556–1.814	0.989
Education	1.053	0.963–1.151	0.258
Marital status			
Married	1.000	Reference	0.411
Unmarried	1.415	0.470–4.257	0.537
Divorced or widowed	2.034	1.302–5.053	0.038
Monthly income			
≤ 3,000	1.000	Reference	0.001
3,000–5,000	0.314	0.169–0.586	< 0.001
>5,000	0.734	0.300–1.793	0.497
Duration of illness ( ≤ 12 months)	1.112	0.620–1.994	0.722
Drug resistance	3.015	1.070–6.348	0.033
T Lymphocyte counts			
CD3	1.000	0.998–1.002	0.956
CD4	1.000	0.996–1.004	0.873
CD8	0.999	0.998–1.001	0.443
CD4/CD8	1.410	0.833–2.387	0.201
Inflammatory indexes			
NLR	1.088	0.942–1.257	0.250
MLR	0.827	0.271–2.517	0.737
PLR	0.999	0.996–1.002	0.390
SII	1.001	1.000–1.020	0.001
Constant	0.028		0.002

NLR, neutrophil/lymphocyte ratio; MLR, monocyte/ lymphocyte ratio; PLR, platelets/lymphocyte ratio; SII, platelets X neutrophils/lymphocytes; *P* < 0.05.

## Discussion

In the present study, more than half (52.1%) of the patients showed depression or/and anxiety to varying degrees, similar to most studies with a prevalence ranging from 40 to 60% (29–31); however, < 60–80% of multidrug-resistant and extensively drug-resistant TB (32). Previous research has shown that *Mtb* infection and mental illness can interact, with an underlying biopsychosocial mechanism that comprises biological factors like the inflammatory cascade and psychosocial factors like perceived stigma (33). Infection-triggered perturbation of the immune system could induce psychopathology, promoting susceptibility to psychiatric symptoms (13). A higher incidence of drug resistance was reported among patients with depression or anxiety compared to those without the symptoms (10.8 vs. 1.9%). Moreover, 38.5% of patients reported depression and anxiety symptoms as comorbidity, in line with other studies reporting a co-occurrence prevalence of 20–50% or above (26, 34, 35). The complex conditions in a large number of critical patients was a possible cause of the high comorbidity of depression and anxiety.

In logistic regression analysis, increasing age, absence of spouse (divorced or widowed), lower income (monthly income ≤ 3,000 yuan), and drug resistance were predictors for developing depression and/or anxiety in tuberculosis patients. TB was more prevalent in older people and there was a progressive increase in the notification rate and mortality with age (36, 37). An increase in depression or anxiety among the elderly is possibly owing to their poorer adaptability, physical deterioration, higher frailty, and decreased social activities. Moreover, divorced or widowed individuals have a greater susceptibility to psychological issues, potentially because this population has experienced more stressful life events and lacks the companionship, comfort, and support from their spouses. Previous studies have also concluded that psychological distress was common in older adults, with ~30% of this population experiencing depressive or anxiety symptoms (38), and the percentage was higher in divorced or widowed population than those married (39). Poverty is both a risk factor and a consequence of TB. Another risk factor for developing depression or anxiety was lower-income ( ≤ 3,000 yuan), potentially due to a lack of welfare security and poorer social support. Consistent with prior studies conducted in China, income and marital status were still key predictors of depression in patients with TB after the socio-demographic variables were controlled (40). In addition, patients with drug resistance need a more complicated regimen along with more critical side effects, longer and worse treatment, and higher costs than those without resistance; these may further contribute to the development of depression and anxiety.

Further, when TB patients with depression and/or anxiety were compared to those without symptoms, they presented lower cell immune function and a stronger inflammatory response. Effective cell-mediated immunity is essential for the control of *Mtb* infection, determining whether the bacterial infection will be controlled or develop into a serious disease. Antigen-specific CD4 T cell response is the primary constituent in the host adaptive immune response against *Mtb*, and CD8 T cells directly eradicate mycobacteria and lyse infected host cells to generate cytokines like IFN-γ against *Mtb* (41). Chronic stress and *Mtb* infection have been considered to reduce immunity, such as decreased peripheral lymphocyte amount and impaired neutrophil phagocytosis. Moreover, the decreased CD4 and CD8 lymphocyte counts have been recognized to be tightly related to depressive and anxiety severity (10). The possible pathological mechanism is that mitochondrial fission caused by stress leading to purine metabolic disturbance in peripheral CD4 T cells can induce anxiety-like behaviors, indicating that CD4 T cells play an essential role in mediating mood disorders (42). In line with this, our study found that CD3, CD4, and CD8 levels were lower in patients with depression and/or anxiety than in those without symptoms, possibly involving in change of psychoneuroimmunology.

Pro-inflammatory factors released by peripheral immunocytes (e.g., lymphocytes, monocytes, neutrophils) can drive neuroinflammation by activating astrocytes and microglia, thus modulating the brain areas participating in regulating mood. Typically, they also reduce the intracerebral monoamine contents, activate neuroendocrine responses, promote excitotoxicity (elevated glutamate contents) and impair brain plasticity (43). An increased count of neutrophils represents inflammatory response intensity, while the decreased count of lymphocytes indicates the impairment of the immune system. Platelets contribute to coagulation and preserve various pro-inflammatory molecules modulating the immune and inflammatory responses (19). The derived indices of peripheral cells especially SII can provide a more comprehensive and stable inflammation assessment since it add platelet counts to NLR formulae to reflect different immune pathways and is less influenced by mixed conditions (44). In general, higher SII reveals enhanced inflammatory response and decreased immune response in patients. Previous studies have confirmed that the NLP and platelet counts were significantly elevated in the active TB group (45, 46). Prior studies demonstrate that SII is in a positive relationship with follow-up depression and anxiety scores (13), and the reduction degree of SII between admission and discharge forecasts the relief of depression among coronavirus disease survivors (47). In a study by HU, increased SII is significantly correlated with post-stroke depression and possibly provides several prognostic clues for early discovering post-stroke depression (44). In TB patients with mood problems, we found elevated peripheral blood inflammatory cells and their count-derived indices (NLR, MLR, PLR, and SII). Furthermore, higher SII was also a predictor of depression and anxiety in TB patients, highlighting more consistent associations of inflammation with mental disorders. The blood routine test is a must-check examination for all hospitalized patients. Peripheral blood cell count-derived indices, particularly SII show the prospect of being widely applied in clinical practice because of their convenience, economy and easily accessibility.

The present study has some limitations. First, the study is cross-sectional, which does not support an explanation for causality. Additionally, no distinction was made in TB type, therefore, potential inaccuracies could exist in data reporting. Lastly, analyzing the same indicators again at different times and exploring the correlation with depression or anxiety levels can improve the accuracy of matching markers.

## Conclusion

The study reports depression and anxiety are prevalent in in-patients with TB. Close attention to the screening and treatment for psychological stress are important to patients with TB owing to its negatively impact on outcomes. We also found lower and higher concentrations of immune and inflammatory parameters in TB patients with depression or anxiety. The dysfunction of the immune-inflammation process among patients with depression or anxiety is stressed; particularly SII has a potential application value in guiding the evaluation and management of TB-related depression or anxiety. However, the time relationship between the indicators and psychopathology should be evaluated, and the true clinical effect of such markers should be confirmed.

## Data availability statement

The data that support the findings of this study are available from the corresponding author upon reasonable request.

## Ethics statement

The studies involving human participants were reviewed and approved by the Biomedical Research Ethics Committee of West China Hospital, Sichuan University. Written informed consent to participate in this study was provided by the participants' legal guardian/next of kin.

## Author contributions

XL and XT designed and supervised the study. XB, HL, and QY all participated in data collection. RR, YZ, and JH provided guidance on study design and methods. XL contributed to data analysis and writing the manuscript. LT and XT assisted in this article revision and writing. All authors contributed substantially to the study design, data interpretation, and the writing of the manuscript. All authors read and approved the final version of the article.

## Funding

This work was supported by the Ministry of Science and Technology of the People's Republic of China (2021ZD0201900) and the National Natural Science Foundation of China (82120108002 and U21A20335).

## Conflict of interest

The authors declare that the research was conducted in the absence of any commercial or financial relationships that could be construed as a potential conflict of interest.

## Publisher's note

All claims expressed in this article are solely those of the authors and do not necessarily represent those of their affiliated organizations, or those of the publisher, the editors and the reviewers. Any product that may be evaluated in this article, or claim that may be made by its manufacturer, is not guaranteed or endorsed by the publisher.

## Abbreviations

TB: tuberculosis
PHQ-9: nine-item Patient Health Questionnaire
GAD-7: seven-item Generalized Anxiety Disorder Scale
*Mtb*: Mycobacterium tuberculosis
SII: systemic immune-inflammation index
NLR: neutrophil/lymphocyte ratio
MLR: monocyte/lymphocyte ratio
PLR: platelets/lymphocyte ratio
SD: standard deviations
DA: depression or anxiety
NDA: neither depression nor anxiety
BMI: body mass index.
